# Antimicrobial Susceptibility and Targeted Molecular Detection of Methicillin Resistance Determinants in *Staphylococcus* spp. Isolated from Broiler BCO Lesions

**DOI:** 10.3390/antibiotics15060606

**Published:** 2026-06-14

**Authors:** Woro Wulandari Kalanjati, Chrystalee Ailani Alvarez, Anh Dang Trieu Do, Adnan Ali Khalaf Alrubaye

**Affiliations:** 1 Department of Poultry Science, University of Arkansas, Fayetteville, AR 72702, USA; worok@uark.edu; 2 Directorate of Risk Management, Animal Quarantine, Indonesian Quarantine Authority, Jakarta 12550, Indonesia; 3 Department of Biology, The University of Texas Rio Grande Valley, Edinburg, TX 78539, USA; chrystalee.alvarez01@utrgv.edu; 4 Cell and Molecular Biology Program, University of Arkansas, Fayetteville, AR 72701, USA; ad086@uark.edu

**Keywords:** BCO, broilers, *Staphylococcus* spp., antimicrobial resistance, β-lactams, *mec*A, *mec*C, disk diffusion

## Abstract

Background/Objectives: Antimicrobial resistance (AMR) in *Staphylococcus* spp. associated with poultry production is an emerging concern with implications for animal and public health. This study aimed to characterize antimicrobial susceptibility patterns and detect targeted methicillin resistance determinants in *Staphylococcus* isolates recovered from broiler chickens affected by bacterial chondronecrosis with osteomyelitis (BCO). Methods: A total of 200 bacterial isolates were evaluated, of which 167 were confirmed as *Staphylococcus* spp. Species identification was performed using presumptive phenotypic characterization followed by 16S rRNA gene sequencing. Antimicrobial susceptibility was assessed using disk diffusion, while presumptive methicillin-resistant phenotypes were evaluated using oxacillin screening and CHROMagar MRSA. Targeted molecular detection of *mec*A and *mec*C was performed by PCR. Results: The isolates demonstrated substantial species diversity, with *S. aureus* as the predominant species. Antimicrobial resistance was mainly observed against β-lactam antibiotics, particularly penicillin (33.5%), whereas high susceptibility was retained for non-β-lactam agents, including ciprofloxacin, tetracycline, trimethoprim–sulfamethoxazole, and azithromycin. A targeted PCR detected *mec*A in 7.2% of isolates, while *mec*C was not detected. The detection of *mec*A in oxacillin-susceptible isolates suggested genotype–phenotype discordance. Conclusions: BCO-associated *Staphylococcus* spp. from broiler chickens showed diverse species distribution, penicillin-dominant resistance, and targeted *mec*A detection across multiple species, supporting the use of combined phenotypic and molecular approaches for methicillin resistance surveillance.

## 1. Introduction

The global demand for affordable animal protein has positioned poultry production as one of the fastest-growing and most economically significant sectors of modern agriculture. In the United States alone, the total poultry industry generated approximately $70.2 billion in value in 2024, of which broiler production accounted for about $45.4 billion, underscoring its dominant contribution to major agricultural regions such as Arkansas, Georgia, and Alabama [1]. Maintaining productivity in this sector is therefore critical for ensuring supply chain stability and meeting increasing global protein demand [2]. However, the intensification required to sustain this level of production has introduced complex biological challenges, including increased disease pressure, environmental stress, and concerns related to antimicrobial resistance (AMR) [3,4].

Over the past two decades, advances in genetic selection, nutrition, and management practices have substantially improved growth rates and feed efficiency in broiler chickens [5,6]. However, these improvements have also increased physiological stress associated with rapid body mass accretion and high-density rearing conditions, contributing to skeletal disorders and increased susceptibility to infection [7]. Among these, bacterial chondronecrosis with osteomyelitis (BCO) has emerged as a leading cause of lameness in fast-growing broilers and represents a major welfare and economic concern [8]. BCO is characterized by bacterial colonization and necrosis of the proximal femur, tibia, and vertebral growth plates and is widely considered a multifactorial disease involving host physiology, mechanical stress, vascular compromise and microbial opportunism [9,10].

*Staphylococcus* spp. are frequently isolated from BCO lesions and are considered key contributors to disease development [11]. BCO-associated *Staphylococcus* spp. represents a phylogenetically diverse group, encompassing *S. aureus*, coagulase-negative staphylococci (CoNS), and other opportunistic species, whose relative contributions may vary according to host condition, flock management, and geographic context [10,11]. These opportunistic pathogens can enter the bloodstream, localize in mechanically compromised bone tissue, and establish persistent infection [11]. Their clinical significance is further amplified by a well-recognized capacity to acquire and maintain AMR determinants, thereby enhancing survival under antimicrobial pressure [12].

Beyond their direct role in BCO pathogenesis, poultry-associated *Staphylococcus* spp. have broader animal health and One Health implications. Staphylococcal infections, including BCO, septicemia, arthritis, and tenosynovitis, have been associated with mortality, culling, impaired growth performance, and carcass condemnation, resulting in economic losses to the poultry industry [8,9,11,13]. In addition, *Staphylococcus* spp. possess virulence mechanisms that support persistence, immune evasion, and tissue invasion [10,12,14]. Certain strains, particularly coagulase-positive staphylococci such as *Staphylococcus aureus*, can survive within host phagocytic cells, evade immune clearance, and contribute to chronic or recurrent infection [15,16].

The presence of antimicrobial-resistant *Staphylococcus* spp. in broiler production systems is also concerning because these organisms may serve as reservoirs of resistance determinants that can circulate across animal, environmental, and human interfaces. In poultry-associated staphylococci, resistance genes may be mobilized by plasmids, transposons, and staphylococcal cassette chromosome *mec* (SCC*mec*) elements, facilitating horizontal gene transfer within and across *Staphylococcus* populations [14]. Previous studies have reported antimicrobial-resistant *S. aureus* and other staphylococci across broiler production and processing environments. For example, *S. aureus* isolates from commercial broiler chickens in South Africa showed high resistance to kanamycin (79.3%), cefoxitin (76.0%), and tetracycline (69.0%), with all isolates classified as multidrug-resistant [17]. In another study, *S. aureus* was detected along the broiler slaughter and processing chain, with contamination rates of 40.0% during defeathering, 26.7% during segmentation, 21.9% during pre-cooling, and 15.6% during storage [18]. These findings support the need for integrated One Health surveillance to better understand the occurrence and spread of antimicrobial-resistant *Staphylococcus* spp. in poultry systems.

## 2. Results

### 2.1. Presumptive Phenotypic Characterization and Species Identification of Staphylococcus spp. Isolates

All isolates were catalase-positive and exhibited presumptive phenotypic characteristics consistent with *Staphylococcus* spp., as determined by biochemical assays and growth on selective and differential media. Growth on MSA indicated salt tolerance, with mannitol-positive isolates producing yellow colonies or yellow discoloration of the medium, while non-mannitol fermenters remained pink to red. On blood agar, colony morphology and hemolytic activity were assessed, with β-hemolysis indicated by clear zones surrounding the colonies. CHROMagar *Staphylococcus* supported presumptive differentiation of staphylococcal isolates based on colony color, while growth on CHROMagar MRSA indicated presumptive methicillin-resistant phenotypes.

However, because phenotypic characteristics may vary among staphylococcal species, final species-level identification was confirmed by 16S rRNA gene sequencing. Among the 167 confirmed *Staphylococcus* isolates, a total of 13 species were identified (Table 1). *S. aureus* was the predominant species, accounting for 29.3% of isolates, followed by *S. cohnii* (24.0%) and *S. saprophyticus* (10.8%). Other species detected at moderate frequencies included *S. kloosii* (9.0%), *S. gallinarum* (7.2%), and *S. lentus* (6.0%), while the remaining species were identified at relatively low proportions (≤4.2% each).

### 2.2. Antimicrobial Susceptibility Patterns of Staphylococcus spp.

The antimicrobial susceptibility profiles of the *Staphylococcus* isolates are presented in Table 2. Resistance was most frequently observed for penicillin (33.5%), whereas the majority of isolates remained susceptible to most of the tested antimicrobial agents. High susceptibility rates were observed for ciprofloxacin, tetracycline, trimethoprim–sulfamethoxazole, and azithromycin, with all isolates classified as susceptible. Intermediate susceptibility was observed primarily for erythromycin (28.1%) and, to a lesser extent, for chloramphenicol (13.8%), reflecting variability in response to these agents. Overall, resistance was largely confined to β-lactam antibiotics, particularly penicillin.

### 2.3. Detection of mec Genes

PCR-based molecular screening was performed to detect methicillin resistance determinants, including *mec*A (universal, classical, and variant targets) and *mec*C, among *Staphylococcus* spp. isolates (*n* = 167). The *mec*A universal and classical targets were each detected in 12 isolates (7.2%), while the *mec*A variant target was identified in 4 isolates (2.4%). No isolates were positive for the *mec*C gene (Table 3). The detected *mec*A genes were primarily associated with *S. aureus* and *S. cohnii*, with additional occurrence in *S. lentus*, *S. saprophyticus*, and *S. argenteus*, indicating that *mec*A is distributed across multiple *Staphylococcus* species rather than being restricted to a single species.

## 3. Discussion

This study provides baseline evidence that BCO-associated *Staphylococcus* spp. in broiler chickens are taxonomically diverse and include both *S. aureus* and multiple coagulase-negative *Staphylococcus* species. The identification of 13 species among 167 confirmed isolates indicates that BCO lesions are not associated with a single dominant staphylococcal taxon, but instead reflect a broader microbial population capable of colonizing compromised skeletal tissue. This finding is consistent with the multifactorial nature of BCO, in which mechanical stress, vascular compromise, host susceptibility, and opportunistic bacterial invasion interact during disease development [8,19].

The predominance of *S. aureus* among the recovered isolates, accounting for 29.3% of the isolates, is biologically consistent with its recognized role as an opportunistic pathogen capable of tissue invasion, immune evasion, and persistence in host tissues. However, the high representation of coagulase-negative staphylococci, particularly *S. cohnii*, *S. saprophyticus*, *S. kloosii*, *S. gallinarum*, and *S. lentus*, is also important because these organisms are increasingly recognized as opportunistic pathogens and reservoirs of antimicrobial resistance determinants [20]. Therefore, limiting surveillance to *S. aureus* alone may underestimate the broader contribution of non-aureus *Staphylococcus* species to poultry-associated infections and resistance ecology.

Phenotypic antimicrobial susceptibility testing showed that resistance was primarily associated with β-lactam antibiotics, especially penicillin, whereas susceptibility to most non-β-lactam antimicrobials remained high (Table 2). Specifically, penicillin resistance was detected in 56 isolates (33.5%), while all isolates were susceptible to ciprofloxacin, tetracycline, trimethoprim–sulfamethoxazole, and azithromycin (Table 2). This pattern suggests a relatively narrow resistance profile in the isolate population rather than widespread multidrug resistance. The higher frequency of penicillin resistance may reflect selective exposure to β-lactam antibiotics and the persistence of β-lactam resistance mechanisms in staphylococcal populations [21,22].

A key finding of this study was the detection of *mec*A targets across multiple *Staphylococcus* species (Table 3). Although methicillin resistance is often discussed primarily in the context of *S. aureus*, *mec*A was also detected in non-aureus species, including *S. cohnii*, *S. lentus*, *S. saprophyticus*, and *S. argenteus* (Table 3). This indicates that methicillin resistance determinants in BCO-associated staphylococci were not restricted to *S. aureus*. Because non-aureus *Staphylococcus* species collectively represented the majority of confirmed isolates, this finding suggests that methicillin resistance in BCO-associated staphylococci should not be viewed only as an MRSA-centered concern but also as part of a broader resistance issue involving multiple *Staphylococcus* species [20,23].

The detection of *mec*A in *S. cohnii* is particularly relevant because this species represented the second most abundant taxon in the isolate collection, accounting for 40 of 167 isolates (24.0%) (Table 1) and *mec*A was detected in this species in the present study (Table 3). Its involvement suggests that methicillin resistance determinants may be maintained within relatively common non-aureus *Staphylococcus* populations rather than being confined to less frequent or incidental taxa. This has important implications for AMR surveillance, as programs focused only on MRSA or phenotypically resistant *S. aureus* may miss resistance determinants carried by coagulase-negative staphylococci. Therefore, poultry-associated CoNS should not be treated only as background organisms, but as potential contributors to the resistome of BCO lesions and the broader poultry production environment [20,24].

From a mechanistic perspective, the distribution of *mec*A across multiple staphylococcal species raises the possibility that horizontal gene transfer may contribute to resistance dissemination within poultry-associated microbial communities. The *mecA* gene is commonly associated with mobile genetic elements such as SCC*mec*, and CoNS are recognized as potential reservoirs of these elements [20]. Therefore, the detection of *mecA* in non-*S. aureus* isolates in this study are not merely a taxonomic observation; rather, it suggests that resistance surveillance should consider the broader staphylococcal population as a possible source of transferable methicillin resistance determinants.

Another important implication is diagnostic. Phenotypic assays, including oxacillin screening and CHROMagar MRSA, provide presumptive evidence of methicillin-resistant phenotypes, whereas PCR directly detects genetic determinants such as *mec*A and *mec*C. In this study, oxacillin screening with 2% NaCl identified 8 isolates (4.8%) as resistant and CHROMagar MRSA identified 10 isolates (6.0%) with presumptive methicillin-resistant phenotypes, while PCR detected *mec*A targets across multiple species (Table 3). Because *mec*A is a genetic marker, it cannot be evaluated phenotypically. Therefore, the most accurate interpretation is that presumptive methicillin-resistant phenotypes were compared with targeted molecular detection of methicillin resistance determinants.

The detection of *mec*A in isolates that may not consistently show phenotypic resistance supports the possibility of genotype–phenotype discordance. Such discordance may occur when *mec*A is present but weakly expressed, heterogeneously expressed, or not expressed under routine laboratory conditions. In this context, phenotypic susceptibility testing alone may underestimate the presence of methicillin resistance determinants. This finding supports the use of combined presumptive phenotypic screening and molecular approaches, especially in non-human and poultry-associated staphylococcal populations where resistance expression may differ from classical clinical MRSA patterns [25,26].

The absence of *mec*C in this isolate collection suggests that *mec*C-mediated methicillin resistance was not detected among the BCO-associated *Staphylococcus* spp. examined (Table 3). Although this reduces concern for *mec*C in the present population, continued surveillance remains warranted because the occurrence of methicillin resistance genes can vary by geography, host species, management practices, and antimicrobial exposure history. The combination of detectable *mec*A and absent *mec*C suggests that methicillin resistance determinants in these isolates were primarily associated with *mec*A-related targets [27].

Overall, this study highlights that BCO-associated *Staphylococcus* spp. represent a diverse microbial population, with *S. aureus* as the predominant species but with substantial representation of non-aureus *Staphylococcus* species (Table 1). The antimicrobial susceptibility profile showed resistance mainly concentrated in β-lactam antibiotics, particularly penicillin (Table 2), while targeted molecular screening showed that *mec*A detection extended beyond *S. aureus* into several non-aureus species (Table 3). These findings strengthen the argument that poultry-associated CoNS should be included in AMR surveillance as potential reservoirs of methicillin resistance determinants.

This study has several limitations, including the absence of whole-genome sequencing and gene expression analyses, which would provide deeper insight into resistance mechanisms and regulatory dynamics. Future studies should expand beyond *mec*A/*mec*C detection to include broader resistance determinants, SCC*mec* typing, whole-genome sequencing, resistome profiling, and transcriptomic analysis. Integrating these data with microbiome analyses would provide a more comprehensive understanding of resistome–microbiome interactions and their role in the emergence and dissemination of antimicrobial resistance in poultry systems.

## 4. Materials and Methods

### 4.1. Source of Bacterial Isolates

Clinical specimens consisting of tissue swabs collected from broiler chickens exhibiting lameness associated with BCO were obtained from archived samples maintained at the Alrubaye Microbiology Laboratory, University of Arkansas, Fayetteville, Arkansas, US. The isolates originated from previous BCO investigations [28,29]. Following initial isolation, all isolates were preserved in 40% glycerol and stored at −80 °C until further analysis.

### 4.2. Microbiological Analysis

Archived isolates were revived by subculturing onto Tryptic Soy Agar (TSA) (Becton, Dickinson and Company, Sparks, MD, USA), Columbia CNA agar (Legacy Biological, RPI, Mount Prospect, IL, USA) with 5% sheep blood, Mannitol Salt Agar (MSA) (Becton, Dickinson and Company, Sparks, MD, USA), and CHROMagar *Staphylococcus* (CHROMagar, Paris, France). Plates were incubated at 37 °C for 18–24 h. Single colonies were selected and evaluated using Gram staining, catalase and coagulase testing, DNase testing as a supportive assay for presumptive differentiation of *S. aureus*, and novobiocin susceptibility testing as a supportive assay for presumptive differentiation of *S. saprophyticus* [30]. Because these phenotypic characteristics can overlap among *Staphylococcus* species, particularly among coagulase-negative staphylococci, final species-level identification was confirmed by 16S rRNA gene sequencing.

### 4.3. Antimicrobial Susceptibility Testing

Antimicrobial susceptibility testing was performed using the disk diffusion method in accordance with Clinical and Laboratory Standards Institute (CLSI) M100 (35th edition) guidelines [31]. Bacterial suspensions were adjusted to a 0.5 McFarland standard and inoculated onto Mueller–Hinton agar (Becton, Dickinson and Company, Sparks, MD, USA). Antibiotic disks were evenly applied to the agar surface, and plates were allowed to dry for 3–5 min before incubation at 37 °C for 18–24 h. The antimicrobial agents included in this study were selected to represent a broad range of antibiotic classes commonly used in both veterinary and human medicine, as well as those relevant to *Staphylococcus* spp. infections. The panel included β-lactams (penicillin, oxacillin, cefoxitin) for assessment of methicillin resistance, macrolides (erythromycin, azithromycin), phenicols (chloramphenicol), fluoroquinolones (ciprofloxacin), tetracyclines (tetracycline), folate pathway inhibitors (trimethoprim–sulfamethoxazole), and aminoglycosides (gentamicin). Zones of inhibition were measured and interpreted according to CLSI M100 (35th edition) guidelines [31].

### 4.4. MRSA Screening

Methicillin resistance was evaluated using oxacillin (1 µg) disk diffusion on Mueller–Hinton agar (Oxoid Ltd., Basingstoke, UK) supplemented with 2% NaCl [30]. Plates were incubated at 37 °C for 18–24 h, and inhibition zone diameters were interpreted according to CLSI M100 (35th edition) guidelines [31]. Additionally, isolates were also cultured on CHROMagar MRSA (CHROMagar, Paris, France) and incubated at 35–37 °C for 18–24 h. Presumptive MRSA colonies were identified based on characteristic mauve coloration, according to the manufacturer’s instructions [32].

### 4.5. DNA Extraction and Molecular Identification

Genomic DNA was extracted using the DNeasy Blood & Tissue Kit (QIAGEN, Hilden, Germany) following the manufacturer’s protocol for Gram-positive bacteria [33]. Extracted DNA was stored at −20 °C until further analysis.

### 4.6. Isolate Identification via 16S rRNA Gene Amplification and Sequencing

The V1–V5 region of the 16S rRNA gene was amplified by PCR using a conventional thermal cycler (Bio-Rad, Hercules, CA, USA), as previously described by Perera et al. [11], each 50 μL reaction contained 25 μL of Phusion^®^ High-Fidelity PCR Master Mix (New England Biolabs^®^ Inc., Ipswich, MA, USA), 1.5 μL of dimethyl sulfoxide (DMSO) (New England Biolabs^®^ Inc., Ipswich, MA, USA), 20.5 μL of nuclease-free water, 0.5 μM of each primer, and 2 μL of DNA template. The forward and reverse primer sequences were 5′-AGAGTTTGATCCTGGCTCAG-3′ and 5′-GTGCGGGCCCCCGTCAATTC-3′, respectively. PCR amplification was performed under the following conditions: initial denaturation at 98 °C for 30 s, followed by 35 cycles of denaturation at 98 °C for 10 s, annealing at 71 °C for 30 s, and extension at 72 °C for 30 s. A final extension was conducted at 72 °C for 3 min, followed by an indefinite hold at 4 °C. The amplified products were confirmed by electrophoresis using a 2% agarose gel in 0.5× TBE buffer at 90–120 V. PCR products were purified using Diffinity RapidTip (Diffinity Genomics, West Henrietta, NY, USA). Purified amplicons with DNA concentrations of 40–60 ng/μL, along with primers at 2–10 pmol/μL, were submitted to Eurofins Genomics (Louisville, KY, USA) for DNA sequencing. The resulting sequences were analyzed using Lasergene 18.1 DNASTAR software (DNASTAR, Inc., Madison, WI, USA) and compared with reference sequences available in the National Center for Biotechnology Information (NCBI) database and the Ribosomal Database Project (RDP). Species-level identification was assigned when sequence similarity exceeded 97% compared with reference database entries.

### 4.7. Molecular Screening of Methicillin Resistance Determinants (mecA and mecC)

PCR was performed to detect *mec*A (universal, classical, and variant) and *mec*C genes using gene-specific primers as previously described [24,34]. Amplified products were visualized by agarose gel electrophoresis under UV illumination to confirm gene presence. Primers targeting 16S rRNA (for *Staphylococcus* spp.) and *nuc* (for *S. aureus*) were included for bacterial identification (Table 4), and *S. aureus* ATCC^®^ 29213 (methicillin-susceptible) (ATCC, Manassas, VA, USA) was used as a reference strain. Multiple *mec*A targets were included to enhance detection sensitivity in this initial surveillance of BCO-derived isolates, as genetic variability may lead to underestimation with a single target. The inclusion of *mec*C aimed to screen for its presence and to explore potential epidemiological links with strains reported in humans and cattle within the food production chain.

### 4.8. Statistical Analysis

Data were analyzed using Microsoft Excel (Microsoft Corporation, Redmond, WA, USA). Antimicrobial susceptibility results were summarized as frequencies and percentages. Agreement between phenotypic and genotypic methods for methicillin resistance detection was evaluated using Cohen’s kappa coefficient (κ). The strength of agreement was interpreted according to the Landis and Koch criteria [35], in which κ values were classified as slight (0.00–0.20), fair (0.21–0.40), moderate (0.41–0.60), substantial (0.61–0.80), or almost perfect (0.81–1.00).

## 5. Conclusions

This study demonstrated that BCO-associated *Staphylococcus* spp. from broiler chickens were taxonomically diverse and included both *S. aureus* and multiple coagulase-negative *Staphylococcus* species. Antimicrobial resistance was mainly associated with β-lactam antibiotics, particularly penicillin, while susceptibility to most non-β-lactam antimicrobials remained high. Targeted molecular screening detected *mec*A across multiple *Staphylococcus* species, including non-aureus taxa, whereas *mec*C was not detected. The presence of *mec*A in isolates with limited or inconsistent phenotypic methicillin-resistant profiles suggests genotype–phenotype discordance and highlights the limitation of relying solely on presumptive phenotypic screening. These findings support the integration of antimicrobial susceptibility testing with targeted molecular detection to improve methicillin resistance surveillance in poultry-associated *Staphylococcus* spp. within a One Health framework.

## Figures and Tables

**Table 1:** Distribution and relative abundance of *Staphylococcus* species among the isolates (*n* = 167).

Species	*n*	%
*S. aureus*	49	29.3
*S. cohnii*	40	24.0
*S. saprophyticus*	18	10.8
*S. kloosii*	15	9.0
*S. gallinarum*	12	7.2
*S. lentus*	10	6.0
*S. arlettae*	7	4.2
*S. argenteus*	4	2.4
*S. sciuri*	4	2.4
*S. pseudoxylosus*	3	1.8
*S. nepalensis*	2	1.2
*S. simulans*	2	1.2
*S. shinii*	1	0.6

Note: *n*, number of isolates; %, percentage of total confirmed *Staphylococcus* isolates.

**Table 2:** Antimicrobial susceptibility profiles of *Staphylococcus* spp. isolates (*n* = 167).

Antimicrobial Agent	S *n* (%)	I *n* (%)	R *n* (%)
β-lactam antibiotics			
Penicillin	111 (66.5%)	0 (0.0%)	56 (33.5%)
Oxacillin	166 (99.4%)	0 (0.0%)	1 (0.6%)
Cefoxitin	166 (99.4%)	0 (0.0%)	1 (0.6%)
Macrolides			
Erythromycin	117 (70.1%)	47 (28.1%)	3 (1.8%)
Azithromycin	167 (100%)	0	0
Phenicols			
Chloramphenicol	139 (83.2%)	23 (13.8%)	5 (3.0%)
Fluoroquinolones			
Ciprofloxacin	167 (100%)	0	0
Tetracyclines			
Tetracycline	167 (100%)	0	0
Folate pathway inhibitors			
Trimethoprim–sulfamethoxazole	167 (100%)	0	0
Aminoglycosides			
Gentamicin	166 (99.4%)	0	1 (0.6%)

Note: S, susceptible; I, intermediate; R, resistant. Antimicrobial susceptibility was interpreted according to CLSI M100 (35th edition) guidelines.

**Table 3:** Distribution of *mec*A gene among *Staphylococcus* spp. isolates (*n* = 167).

Gene Target	Positive *n* (%)	Species Detected
*mec*A (universal)	12 (7.2%)	*S. aureus* (4/12), *S. Cohnii* (5/12), *S. Lentus* (1/12), *S. Saprophyticus* (1/12), *S. argenteus* (1/12)
*mec*A (classical)	12 (7.2%)	*S. aureus* (5/12), *S. cohnii* (5/12), *S. lentus*(1/12), *S. saprophyticus* (1/12)
*mec*A (variant)	4 (2.4%)	*S. aureus* (2/4), *S. cohnii* (1/4), *S. argenteus* (1/4)
*mec*C	0 (0.0%)	Not detected

**Table 4:** Primer used for identification of *Staphylococcus* spp. in this study.

Target Gene	Sequence (5′–3′)	Annealing Temperature (°C)	Amplicon Size (bp)	Reference
*nuc* *	GCGATTGATGGTGATACGGT AGCCAAGCCTTGACGAACTAAAGC	55 °C 30 s	147 bp	[34]
Universal *mec*A	ACG TTA CAA GAT ATG AAG ACA TTA ATA GCC ATC ATC	55 °C 1 min	574 bp	[24]
Variant *mec*A	CAG GCA TGC AGA AAA ATC AA TTG AGT CGA ACC AGG TGA TG	55 °C 1 min	809 bp	
Classical *mec*A	AAA ATC GAT GGT AAA GGT TGG C AGT TCT GCA GTA CCG GAT TTG C	55 °C 30 s	533 bp	
*mec*C	GAA AAA AAG GCT TAG AAC GCC TC GAA GAT CTT TTC CGT TTT CAG C	50 °C 1 min	718 bp	

* The table lists all primers used in this study. The *nuc* gene was used for confirmation of *S. aureus*, while *mec*A and *mec*C were used to detect methicillin resistance genes.

## Data Availability

The data presented in this study are available from the corresponding author upon reasonable request.

## Abbreviations

The following abbreviations are used in this manuscript:

AMR	Antimicrobial resistance
BCO	Bacterial chondronecrosis with osteomyelitis
CLSI	Clinical and Laboratory Standards Institute
DNA	Deoxyribonucleic acid
MDR	Multidrug resistance
MHA	Mueller–Hinton agar
MRSA	Methicillin-resistant *Staphylococcus aureus*
PCR	Polymerase chain reaction
cPCR	Conventional polymerase chain reaction
PBP	Penicillin-binding protein
PBP2a	Penicillin-binding protein 2a
SCC*mec*	Staphylococcal cassette chromosome *mec*
TSA	Tryptic soy agar
MSA	Mannitol salt agar
OS-MRSA	Oxacillin-susceptible *mec*A-positive *Staphylococcus aureus*
S	Susceptible
I	Intermediate
R	Resistant
Κ	Cohen’s kappa coefficient
